# Bioprocessed Production of Resveratrol-Enriched Rice Wine: Simultaneous Rice Wine Fermentation, Extraction, and Transformation of Piceid to Resveratrol from *Polygonum cuspidatum* Roots

**DOI:** 10.3390/foods8070258

**Published:** 2019-07-15

**Authors:** Kai-Ruei Yang, Hui-Chuan Yu, Chun-Yung Huang, Jen-Min Kuo, Cheng Chang, Chwen-Jen Shieh, Chia-Hung Kuo

**Affiliations:** 1 Department of Seafood Science, National Kaohsiung University of Science and Technology, Kaohsiung 811, Taiwan; 2 Biotechnology Center, National Chung Hsing University, Taichung 402, Taiwan

**Keywords:** rice wine, piceid, resveratrol, *Polygonum cuspidatum*, antioxidant activity, ultrafiltration, clarification

## Abstract

A new bioprocess to produce resveratrol-enriched rice wine was established and the effects of adding *Polygonum cuspidatum* root powder to rice wine fermentation were investigated. In this new process, piceid and resveratrol were extracted from *P. cuspidatum* roots to rice wine and piceid was converted to resveratrol by β-glucosidase during fermentation. After 10 days co-fermentation, rice wine with high levels of resveratrol was obtained, which contained ~14% (*v*/*v*) ethanol, 122 mg/L piceid, and 86 mg/L resveratrol. The resveratrol-enriched rice wine had enhanced antioxidant activity with significantly stronger 2,2-diphenyl-1-picrylhydrazyl (DPPH) radical scavenging activity, ferric ion reducing power, and ferrous ion chelating capability. Ultrafiltration (UF) was employed in this study using hollow fibers to clarify the end product, increase shelf life without heat treatment, and maintain the quality of the phenolic compounds. The boiled and UF-treated rice wine were evaluated for ethanol, piceid, resveratrol, clarity, aerobic plate count, total acidity, pH, reducing sugars, and amino acids. The quality of the resveratrol-enriched rice wine was maintained after four weeks storage at normal refrigeration temperatures.

## 1. Introduction

Resveratrol (3,5,4-trihydroxystilbene) is a polyphenol that exists in several plants, including bilberry, blueberry, cranberry, grape, and peanut [1,2,3]. Resveratrol possesses physiological functions reported to inhibit the migration and metastasis of cancer cells [4], as well as antioxidative, anti-melanoma [5], fatty-liver-reducing [6], anti-obesity [7], anti-inflammatory, cardioprotective, neuroprotective, and antitumor properties [8]. Resveratrol from grape juice [9], grape seeds [10], grape skins [11], and red wine [12] has been extensively studied. It is generally agreed that moderate consumption of red wine can prevent cardiovascular disease. The resveratrol in red wine is believed to be a key molecule since it inhibits low-density lipoprotein oxidation and platelet aggregation in blood [13,14]. Resveratrol is mainly present in grape skins and its concentration in grape pulp is low or absent altogether. Red wine is made from pomace and fermentation occurs together with grape skins, which gives the wine higher amounts of resveratrol than white wine [15,16]. Despite this, resveratrol concentration in red wine is relatively low, ranging from around 1.6 to 3.6 mg/L [17].

*Polygonum cuspidatum* is a perennial plant belonging to the Polygonaceae family. Dried *P. cuspidatum* roots, also called Hu Zhang in Chinese, are herbal medicine for the treatment of cough, arthralgia, chronic bronchitis, jaundice, amenorrhea, hypertension, and hypercholesterolemia [18]. The main phenolic compounds extracted from *P. cuspidatum* roots have been identified as resveratrol, piceid, and emodin [19]. Emodin has been shown to possess anti-inflammatory, antibacterial, antiglycation, and antineoplastic activities [20,21,22,23]. However, emodin is more hydrophobic and only small amounts of emodin can be extracted at low ethanol concentrations compared to piceid and resveratrol [24]. In plants, resveratrol is usually present in piceid (resveratrol 3-β-mono-D-glucoside), its glycoside derivative. Extracts from *P. cuspidatum* roots contain high amounts of piceid, which can be converted to resveratrol when treated with β-glucosidase or cellulase or fermented by *Aspergillus oryzae* [25,26,27]. Rice wine is an alcoholic beverage made from rice, traditionally consumed in East Asia, Southeast Asia, and South Asia [28]. Qu is usually used as a starter in rice wine fermentation [29]. Qu is rich in a wide variety of microorganisms, such as the filamentous molds *A. oryzae* and *Rhizopus oryzae* and the amylolytic yeast *Saccharomycopsis fibuligera*, as well as various enzymes, including amylase, glucoamylase, protease, and phosphatase [30,31]. *A. oryzae* and *R. oryzae* are responsible for producing amylase during fermentation; these two microorganisms can also produce β-glucosidase [32,33]. 

**Figure 1:**
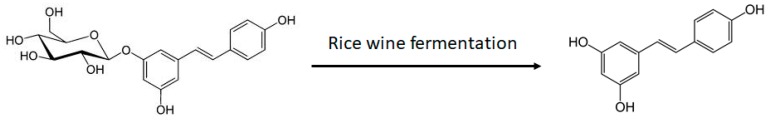
Scheme representing transformation of piceid to resveratrol during rice wine fermentation.

Functional beer, a new product recently obtained via co-fermentation with medicinal herbs, has been commercialized in Japan [34], which has prompted interest in developing a high value rice wine with health benefits. Most medicinal herbs are rich in antioxidants, especially phenolic compounds. Therefore, wines made from medicinal herbs are rich in natural antioxidants to enhance the health functions of fermented wines. In recent years, resveratrol has become a candidate nutritional substance for cancer prevention widely available as a botanical dietary supplement. To date, *P. cuspidatum* roots are used as a commercial source of resveratrol. However, the conversion of excessive piceid in *P. cuspidatum* to resveratrol is still necessary. As such, the addition of *P. cuspidatum* roots during rice wine fermentation to produce resveratrol-enriched rice wine was investigated. In this new process, as depicted in Figure 1, resveratrol can be extracted and piceid converted to resveratrol by β-glucosidase during fermentation.

Sterilization by boiling is commonly used to kill microorganisms and deactivate the enzymes in rice wine to protect it against rancidity and deterioration. However, boiling at high temperatures might increase turbidity, cause heavy browning, or decompose bioactive compounds. Ultrafiltration (UF) is a new technology widely used by the food industry in recent years to remove enzymes, microorganisms, and turbidity contents. The separation principle of the UF membrane is based on mechanical filtration, driven by the pressure difference between the internal and external sides of the membrane [35,36,37,38]. Compared to boiling, the advantages of UF are no phase changes, operation at low temperatures, low energy consumption, simple operation, and better preservation of the original flavor and nutrients. At present, many studies use ultrafiltration for juice clarification [39,40], but the application of UF in wine sterilization is still limited.

The objective of this study was to develop a new brewing process for resveratrol-enriched rice wine. The effects of *P. cuspidatum* on the composition characteristics of rice wine were investigated. The antioxidant activity of rice wine was determined based on the scavenging activity of 2,2-diphenyl-1-picrylhydrazyl (DPPH) radicals, ferrous ion chelating activity, and reducing activity. Finally, ultrafiltration was employed to remove enzymes, microorganisms, and turbidity contents from the resveratrol-enriched rice wine.

## 2. Materials and Methods 

### 2.1. Materials

Polished rice (11.9% moisture, 82.4% carbohydrate, 5.7% protein, and 0% lipid) from the 2017 crop was purchased from the Hua-Tung Rice Co., Ltd. (Hualien, Taiwan). Dried *P. cuspidatum* roots were purchased from local Chinese herbal medicine stores and ground into powder with ~0.62 mm particles. Qu was purchased from Yong Xin Jiuqu Co., Ltd. (Changhua, Taiwan). Piceid and *p*-nitrophenyl-β-D-glucopyranoside (PNG) was purchased from Sigma-Aldrich (MO, USA). Resveratrol was purchased from Changsha Nutramax Biotechnology (Changsha, China). DPPH (2,2-Diphenyl-1-picrylhydrazyl) and *o*-phthaldialdehyde were purchased from Alfa Aesar (Tewksbury, MA, USA). Finally, 3,5-dinitrosalicylic acid and ferrozine were purchased from Acros (Morris Plains, NJ, USA). Unless otherwise noted, all reagents and chemicals were of analytical grade.

### 2.2. Rice Wine Production 

Rice and water at a ratio of 1:1 were steam-cooked for 40 min. The moisture content of the cooked rice was 45.42%. After cooling to 30 °C, Qu was added to the rice at a ratio of 1:200 (Qu: steam-cooked rice; *w*/*w*) and mixed well. Then, 100 g of the mixture was added to a 500 mL screw capped glass jar and the mixture was cultured at 30 °C for 2 days. Varying amounts of *P. cuspidatum* root powder (1%, 3%, or 5% based on the weight of the steam-cooked rice) and 100 mL sterile water were then added to the jar for simultaneous extraction and transformation of piceid to resveratrol from *P. cuspidatum* root powder during rice wine fermentation. Ethanol, piceid, and resveratrol concentrations were measured periodically during fermentation.

### 2.3. Clarification and Sterilization by Ultrafiltration 

Rice wine fermented with 5% *P. cuspidatum* for 10 days was used for UF processing. The rice wine was centrifuged at 10,000 rpm for 5 min and the supernatant filtered through Whatman No. 1 filter paper. The rice wine was then ultrafiltered by a tangential flow filtration system (MAP-TFF, Lefo Science, Taipei, Taiwan) equipped with a hollow-fiber filter module with a molecular weight cut-off of 3 kDa or 10 kDa (MicroKros, Spectrum Labs, Rancho Dominguez, CA, USA). The rice wine was stored at 4 °C to test its shelf life. Samples were analyzed for ethanol, piceid, and resveratrol concentrations, as well as total acidity, turbidity, reducing sugars, amino acids, and aerobic plate count (APC). The control experiment, sterilization by boiling, was conducted by placing the centrifuged supernatant in a boiling water bath for 20 min. 

### 2.4. Antioxidative Properties of Resveratrol-Enriched Rice Wine

The DPPH radical scavenging activity was measured according to the method of Huang et al. [41], with some minor modifications. Briefly, 0.5 mL of the sample was added to 0.5 mL 0.1 mM freshly prepared DPPH solution (in ethanol). After DPPH radicals transferred hydrogen to antioxidative agents, the solution lightened in color at 517 nm due to the reduction in optical absorbance. The mixture was shaken vigorously for 1 min then left to stand for 30 min in the dark at room temperature. The absorbance of all sample solutions was measured at 517 nm using a UV/VIS spectrophotometer (Hitachi U-2900, Tokyo, Japan). The DPPH radical scavenging activity was calculated using the following equation:

Scavenging activity (%) = (1 − (A_sample_)/A_control_) × 100 (1)

The reducing power assay was measured according to the method of Conde et al. [42], with some minor modifications. Briefly, 1 mL of sample was added to 1 mL of phosphate buffer (200 mM, pH 6.6) and 1 mL of 1% potassium ferricyanide. The solution was allowed to react for 20 min at 50 °C, and then 1 mL of 10% trichloroacetic acid (in ethanol) was added. The reactant was centrifuged at 10,000 rpm for 10 min. Then 0.5 mL of the upper layer of the solution was mixed with 0.5 mL of distilled water and 0.1 mL of 0.1% ferric chloride in test tubes. After 10 min of reaction, the resulting solution was measured at 700 nm. Increased absorbance (A700) of the reaction mixture indicated increased reducing power. The standard curve was linear between 20 and 100 ppm vitamin C. Results are expressed in ppm vitamin C. Additional dilution was needed if the measured absorbance value was over the linear range of the standard curve.

The ferrous ion chelating power was measured according to the method of Wu et al. [43], with some minor modifications. Briefly, 1 mL of sample was added to 0.1 mL of 2 mM ferrous chloride and 3.7 mL of methanol. The reaction was started by the addition of 5 mM ferrozine (0.2 mL), and the mixture was shaken vigorously before being left to stand at room temperature for 10 min. Absorbance of the resulting solution was measured at 562 nm. The chelating power was calculated according to Equation (1).

### 2.5. Analysis

The ethanol concentration was quantified using a Thermo Quest Trace 2000 gas chromatograph equipped with a flame ionization detector and an MXT-WAX capillary column (30 m × 0.28 mm i.d.; film thickness 0.25 μm; RESTEK, Bellefonte, PA, USA). Injector and detector temperatures were set at 200 °C and 250 °C, respectively. Initial temperature of the column oven was set at 40 °C for 2 min, and then increased to 215 °C at 20 °C min^−1^. Pure helium was used as a carrier gas at a flow rate of 0.5 mL min^−1^. Piceid and resveratrol were assayed by injecting 20 μL of the sample into an HPLC system, consisting of a Hitachi L-2130 HPLC pump and a Hitachi L-2420 UV/VIS detector (Hitachi, Tokyo, Japan), using an Inertsil ODS-3 column (5 μM, 250 mm × 4.6 mm). Deionized water and methanol containing 0.1% acetic acid were used for gradient elution from 10 to 100% methanol for 20 min, followed by elution at 100% methanol for 5 min. The flow rate was set at 1.0 mL min^−1^. The UV detector was set at a wavelength of 303 nm. The standard curves for piceid and resveratrol were linear over the range 25–125 μg/mL (*R*^2^ = 0.9987) and 16–80 μg/mL (*R*^2^ = 0.9995), respectively. Piceid and resveratrol levels in the samples were quantified by comparing their retention times and peak areas with those of the standards. Samples were diluted if the concentrations fell outside the standard curve ranges. β-Glucosidase was determined using PNG as the substrate. Briefly, 50 μL of sample was added to 50 μL of 20 mM PNG (in 50 mM, pH 4.5 citric buffer) and was incubated for 30 min at 37 °C. The reaction was stopped by the addition of 0.9 mL of 0.1 M sodium carbonate. The release of *p*-nitrophenol, resulting from the β-glucosidase-catalyzed hydrolysis of PNG, was measured by reading the absorbance at 405 nm (ε405 = 17.0 mM^−1^ cm^−1^). One unit of β-glucosidase activity was defined as the amount of enzyme that liberated 1 μmoL of *p*-nitrophenol per minute. The reducing sugar content of the rice wine was determined by 3,5-dinitrosalicylic acid colorimetry using glucose as the standard [44]. Total acidity of the rice wine was rapidly determined by titrimetry. The amino acid content was determined by *o*-phthaldialdehyde colorimetry using glutamine as the standard [45]. Clarity was determined by measuring the transmittance (T%) at 680 nm using a UV/VIS spectrophotometer (Hitachi U-2900, Tokyo, Japan); distilled water was used as the control.

## 3. Results and Discussion

### 3.1. Rice Wine Fermented with P. cuspidatum 

**Figure 2:**
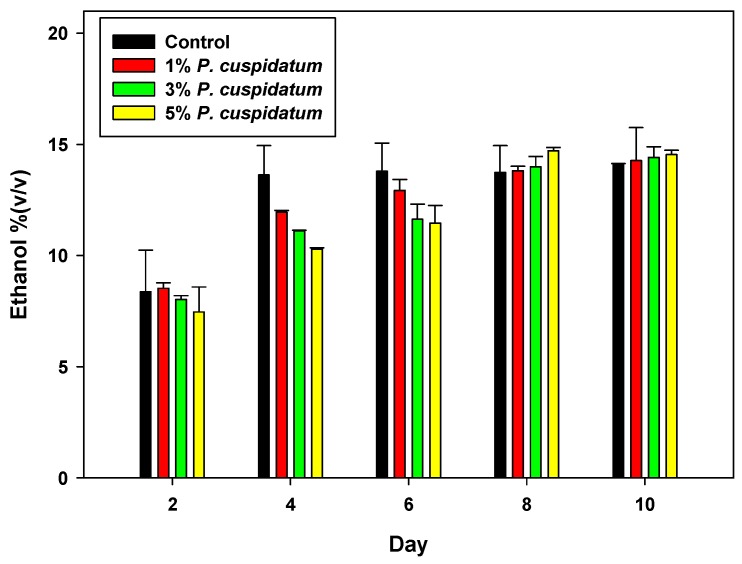
Effect of *P. cuspidatum* roots on ethanol yield during rice wine fermentation. Control was performed without the addition of *P. cuspidatum*.

Steam-cooked rice mixed with Qu was incubated for 2 days in order to induce the enzymes involved in ethanol fermentation, including amylase, glucoamylase, and protease. After incubation, varying amounts of *P. cuspidatum* root powder (1%, 3%, or 5%) were added with sterile water to the mixture for rice wine fermentation and simultaneous extraction of piceid and resveratrol. The ethanol yield during the period of fermentation is shown in Figure 2. The addition of *P. cuspidatum* decreased the initial ethanol production rate for the first 6 days, compared to the control; however, the ethanol yields were the same after 8 d of fermentation. The final ethanol yields were 14.2%, 14.4%, 14.5%, and 14.1%, for rice wine with 1%, 3%, and 5% *P. cuspidatum* and the control, respectively. The results showed that *P. cuspidatum* did not influence ethanol production.

### 3.2. Biotransformation of Piceid to Resveratrol during Rice Wine Fermentation

**Figure 3:**
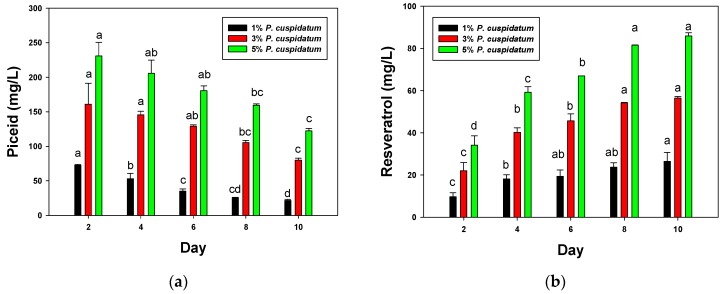
Effect of *P. cuspidatum* roots on the (**a**) piceid and (**b**) resveratrol yields during rice wine fermentation. ^a,b,c,d^ When the bars of the same color have significant differences at *p*-value less than 0.05, homogeneous groups in each variable are identified by the same superscript letter according to the LSD (least significant difference) test.

Piceid and resveratrol, which originated from the *P. cuspidatum* roots, were infused into the rice wine during fermentation. The change in piceid yield during fermentation is shown in Figure 3a. The highest piceid concentrations were found in the first 2 days at 73, 161, and 231 mg/L for wine with 1%, 3%, and 5% *P. cuspidatum*, respectively, but after 10 days, these levels decreased to 22, 80, and 122 mg/L. Resveratrol showed a different trend; the change in its yield during fermentation is shown in Figure 3b. Resveratrol concentration was lowest the first 2 days at 10, 22, and 34 mg/L for wine with 1%, 3%, and 5% *P. cuspidatum*, respectively. Unlike piceid, however, resveratrol concentration increased with fermentation time, with the yield increasing gradually for the first 8 days and then levelling off after 10 days. Wang et al. studied the biotransformation of piceid to resveratrol by *A. oryzae* and reported that the yield of trans-resveratrol first increased, and then decreased after it reached its highest value during fermentation [46]. It has been reported that resveratrol increases the life span of yeast in winemaking [47]. The levelling off in resveratrol concentration after 10 days may be due to the converted resveratrol being consumed by yeast. The highest resveratrol yield was 26, 57, and 86 mg/L after 10 days for wine with 1%, 3%, and 5% *P. cuspidatum*, respectively. These results indicate that piceid was converted to resveratrol during rice wine fermentation. 

**Figure 4:**
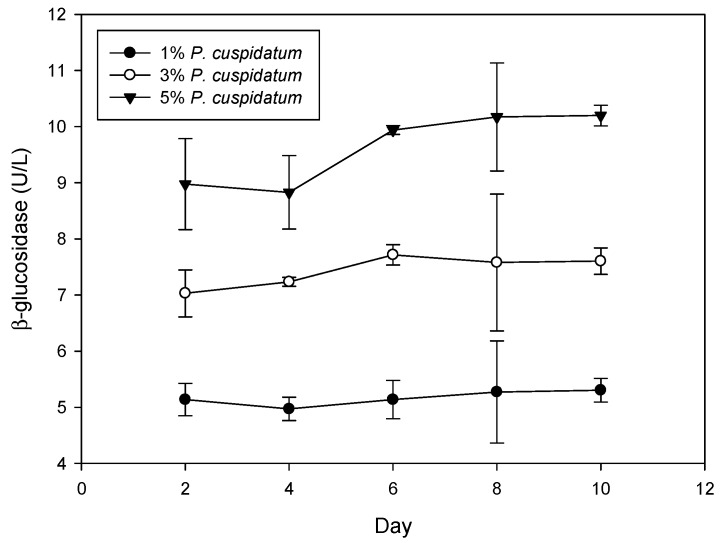
Effect of *P. cuspidatum* roots on the β-glucosidase activity during rice wine fermentation.

β-Glucosidase catalyzes the hydrolysis of β-O-glucosidic linkages between β-D-glucose and an aglycone [48,49]. Therefore, β-glucosidase activity was examined during rice wine fermentation. As shown in Figure 4, β-glucosidase activity increased with the amount of *P. cuspidatum* supplementation. After 10 days fermentation, the β-glucosidase activity was 5.3, 7.6, and 10.2 U/L for wine with 1%, 3%, and 5% *P. cuspidatum*, respectively. The results indicate that β-glucosidase activity was induced by *P. cuspidatum* during fermentation. The mole conversion of piceid to resveratrol was defined as the increase of resveratrol mole per the decrease of piceid mole from days 2 to 10. The mole conversions for wine with 1%, 3%, and 5% *P. cuspidatum* were 56%, 73%, and 82%, respectively. These results indicate that the mole conversion is directly related to β-glucosidase activity. β-Glucosidase from *A. niger* has been used to hydrolyze piceid to produce resveratrol in red wine, which increases resveratrol to 75% [50], but the overall content of resveratrol is still low (~2.8 mg/L).

### 3.3. Antioxidant Capacity of Resveratrol-Enriched Rice Wine

Oxygen is the basic component needed to maintain most life, but oxygen can form reactive oxygen species (ROS) or free radicals, such as superoxide anion radicals, hydroxyl radicals, hydrogen peroxide, singlet oxygen, or nitric oxide [51]. Excessive free radicals or ROS are harmful to the human body because these components are highly chemically reactive and may damage cellular DNA, proteins, nucleic acids, and lipids, thereby destroying the normal functions of cells, tissues, and organs. The importance of anti-oxidative components is due to their ability to reduce the damage to free radical-mediated cells, tissues, and organs within the human body. Polyphenols possess a well-known antioxidant capacity because of their ability to scavenge free radicals, inhibit free radical formation, and chelate with metals to protect cells from oxidative damage and further prevent the occurrence of cancer and cardiovascular disease [52]. Therefore, the aim of this section was to demonstrate the antioxidant capacity of resveratrol-enriched rice wine. 

The DPPH radical scavenging capacities of rice wine, and rice wine fermented with 1%, 3%, and 5% *P. cuspidatum* root were 5.12%, 29.70%, 30.42%, and 37.18%, respectively (Table 1). The DPPH radical scavenging capacity of the rice wine fermented with *P. cuspidatum* was higher than the control. DPPH radical scavenging activity increased with the amount of *P. cuspidatum* supplementation. The higher percentage values of DPPH radical scavenging capacity in rice wine fermented with *P. cuspidatum* indicate that resveratrol-enriched rice wine scavenges free radicals in vitro, consistent with the study results for total amounts of resveratrol. Similarly, the DPPH radical scavenging capacity for 250 mg/L of *P. cuspidatum* extract was 22.22% [5], which is lower than the resveratrol-enriched rice wine. This might be due to the conversion of piceid to resveratrol during rice wine fermentation.

Reducing power is another important indicator of antioxidant activity in natural components. The reducing capacity of a compound may serve as a significant indicator of its potential antioxidant activity and indicates that these compounds are electron donors [53]. The quantification of reducing power is based on the antioxidant’s reaction with potassium ferricyanide (Fe^3+^) to form potassium ferrocyanide (Fe^2+^). This then reacts with ferric chloride to form a ferric ferrous complex that has a maximum absorption at 700 nm. As shown in Table 1, the reducing powers of rice wine, and rice wine fermented with 1%, 3%, and 5% *P. cuspidatum* were 6.93, 107.29, 189.63, and 293.92 ppm vitamin C equivalent, respectively. The reducing power increased with the amount of *P. cuspidatum*, similar to the results of DPPH radical scavenging activity. 

Another antioxidant system was investigated by determining the chelating power of the ferrous ion. Ferrozine can form complexes with the ferrous ion, resulting in a red color. In the presence of chelating agents, complex formation is interrupted, decreasing the red color. The chelation capacity of rice wine increased with the amount of *P. cuspidatum*. Rice wine fermented with *P. cuspidatum* showed higher ferrous ion chelating activity (73.05~80.91%) than control (69.36%). In general, free transition metals like iron react with either hydrogen or lipid peroxides to produce hydroxyl radical compounds and alkoxyl radicals, also known as the Fenton reaction. These radicals are extremely reactive and will significantly accelerate oxidative degradation.

In summary, the antioxidant activity (based on DPPH radical scavenging activity and reducing capacity) of the resveratrol-enriched rice wine was stronger than rice wine.

### 3.4. Clarification and Sterilization of Resveratrol-Enriched Rice Wine

Traditionally, boiling is used to kill microorganisms and deactivate enzymes in rice wine to protect it against rancidity and deterioration. UF is a new technology widely used by the food industry in recent years for juice clarification [54,55], but there are few reports on wine clarification. Macromolecular substances, like microorganisms, enzymes, and turbid substances, cannot pass through the UF membrane and are therefore removed from the rice wine. Aroma components and smaller nutrient molecules that can pass through the UF membrane are retained. The resveratrol-enriched rice wine was ultrafiltered with a tangential flow filtration system equipped with a hollow-fiber filter module with a molecular weight cut-off of 3 kDa or 10 kDa. The effect of the UF process on rice wine quality is shown in Table 2. UF did not affect the ethanol content, total acidity, pH, reducing sugar content, or amino acid content of the rice wine. These parameters were, respectively, about 14% ethanol, 4 g/L total acidity, 3.7 mg/mL reducing sugars, and 1.1 mg/mL amino acids. However, the UF-treated rice wine had better clarity (transmittance at 680 nm was more than 99%) than boiled rice wine (91.4%), as shown in Table 2. The transmittance shows a large difference between the UF-treated and boiled rice wine. Li et al. have shown that suspended solids and juice turbidity can be almost entirely removed by the UF membrane, but the pH, acidity, sugar, and soluble solid content of the juice showed no significant changes [56]. Furthermore, the microbiological characteristics of the UF-treated resveratrol-enriched rice wine were in agreement with food safety regulations, since the number of aerobic plate count was not detected, as shown in Table 2. These results indicate that UF could be an alternative process for clarification and sterilization. UF has been used to clarify nixtamalization waste waters for the recovery of phenolic compounds [57]. In our study, the 3 kDa UF process slightly decreased the amount of piceid and resveratrol, but the concentration of piceid and resveratrol did not change by the 10 kDa UF process. The resveratrol-enriched rice wine obtained from 10 kDa UF contains 120 mg/L piceid and 84 mg/L resveratrol, which is at least 23 times greater than the resveratrol concentration of red wine (~1.6 to 3.6 mg/L resveratrol) [17]. As such, resveratrol-enriched rice wine would have a beneficial effect on physical health.

### 3.5. Storage Study

The UF-treated rice wine was stored for 4 weeks to check its shelf life. Polymerization of phenolic compounds and interactions with other components (e.g., proteins or sugars) can lead to increased turbidity [58,59,60]. The rates of these reactions slow in low temperature conditions. Therefore, the UF-treated rice wine was stored at a lower temperature to keep the turbidity within a minimal range. Various parameters, including ethanol, piceid, and resveratrol levels, clarity, aerobic plate count, total acidity, pH, total reducing sugars, and total amino acids, were determined at the onset and after each consecutive week. As the results in Table 3 show, the quality of rice wine remained almost the same for 4 weeks. The quality indices (ethanol, piceid, and resveratrol) of the UF-treated rice wine showed no significant change after four weeks of storage, and therefore it can be safely asserted that the UF-treated rice wine can be stored for four weeks at normal refrigeration temperature (4 °C) without any additives or preservatives.

## 4. Conclusions

This study established a new process to combine rice wine fermentation, the extraction of phenolic compounds, and the biotransformation of piceid to resveratrol to obtain functional rice wine. This novel resveratrol-enriched rice wine has enhanced antioxidant activities, including increased free radical-scavenging activity, ferric ion reducing power, and ferrous ion chelating activity. The results confirm that UF has great potential for the clarification and sterilization of rice wine as it retains bioactive compounds. This is because UF operates at room temperature, no chemicals are used, no phase changes occur, and it has a high recovery yield. The UF-treated clarified product presents good quality characteristics, and also meets microbiological safety requirements. Therefore, this novel product could be consumed as a resveratrol-rich functional wine or a natural herb-fermented rice wine.

## Figures and Tables

**Table 1:** DPPH free radical scavenging activity, ferrous ion chelating activity and reducing power of resveratrol-enriched rice wine. Control was performed without the addition of *P. cuspidatum*.

Group	DPPH Radical Scavenging Activity (%)	Reducing Power (Vitamin C Equivalent ppm)	Ferrous-Ion Chelating Activity (%)
Control	5.12 ± 2.70 ^c^	6.93 ± 0.20 ^d^	69.36 ± 0.83 ^c^
1% *P. cuspidatum*	29.70 ± 0.11 ^b^	107.29 ± 6.43 ^c^	73.05 ± 1.36 ^b^
3% *P. cuspidatum*	30.42 ± 1.60 ^b^	189.63 ± 6.94 ^b^	79.14 ± 0.61 ^a^
5% *P. cuspidatum*	37.18 ± 6.05 ^a^	293.92 ± 3.47 ^a^	80.91 ± 1.28 ^a^

^a,b,c,d^ When there are significant differences at *p*-value less than 0.05, homogeneous groups in each variable are identified by the same superscript letter according to the LSD test. DPPH: 2,2-Diphenyl-1-picrylhydrazyl.

**Table 2:** Quality characteristics of resveratrol-enriched rice wine treated by boiling and ultrafiltration.

Item	Boiling	Ultrafiltration	
		10 KD	3 KD
Ethanol (%; *v*/*v*)	14.22 ± 0.66	14.40 ± 0.24	14.50 ± 0.92
Piceid (mg/L)	120.10 ± 6.07	120.67 ± 3.05	104.75 ± 2.51
Resveratrol (mg/L)	82.50 ± 2.52	84.93 ± 0.47	80.58 ± 0.43
Clarity (%T)	91.4 ± 0.00	99.35 ± 0.05	99.60 ± 0.05
Aerobic plate count (CFU/mL)	N.D. ^1^	N.D.	N.D.
Total acidity (g/L)	3.83 ± 0.23	4.05 ± 0.00	4.05 ± 0.00
pH	3.66	3.65	3.65
Reducing sugars (mg/mL)	3.59 ± 0.08	4.09 ± 0.19	3.55 ± 0.21
Amino acids (mg/mL)	1.11 ± 0.03	1.04 ± 0.00	1.05 ± 0.02

^1^ Not detected.

**Table 3:** Quality characteristics of resveratrol-enriched rice wine treated by boiling and ultrafiltration process after storage.

Treat	No. of Weeks	Ethanol (%)	Piceid (mg/L)	Resveratrol (mg/L)	Clarity (%T)	APC (CFU/mL)	Total Acidity (g/L)	pH	Reducing Sugars (mg/mL)	Amino Acids (mg/mL)
Boiling	1	14.8 ± 1.9 ^a^	120.9 ± 0.9 ^a^	84.1 ± 2.6 ^a^	89.8 ± 0.1 ^b^	N.D.^1^	3.38 ± 0.2 ^a^	3.60	3.7 ± 0.2 ^b^	1.55 ± 0.02 ^d^
	2	14.8 ± 0.5 ^a^	117.9 ± 8.2 ^a^	85.8 ± 4.1 ^a^	91.0 ± 0.0 ^a^	N.D.	4.05 ± 0.0 ^a^	3.69	4.1 ± 0.1 ^a^	1.97 ± 0.01 ^a^
	3	14.6 ± 0.0 ^a^	113.2 ± 9.3 ^a^	79.1 ± 1.3 ^a^	88.6 ± 0.1 ^c^	N.D.	4.05 ± 0.0 ^a^	3.60	4.0 ± 0.1 ^a^	1.75 ± 0.01 ^b^
	4	14.2 ± 0.3 ^a^	113.4 ± 1.0 ^a^	80.0 ± 2.1 ^a^	90.0 ± 0.0 ^b^	N.D.	4.73 ± 0.7 ^a^	3.60	4.0 ± 0.1 ^ab^	1.67 ± 0.01 ^c^
UF-10K	1	14.1 ± 0.7 ^a^	120.4 ± 8.0 ^a^	83.9 ± 0.8 ^a^	99.3 ± 0.0 ^a^	N.D.	3.60 ± 0.0 ^c^	3.60	4.3 ± 0.2 ^a^	1.48 ± 0.02 ^d^
	2	14.2 ± 0.7 ^a^	122.8 ± 6.5 ^a^	85.2 ± 3.7 ^a^	98.7 ± 0.1 ^c^	N.D.	4.05 ± 0.0 ^b^	3.62	4.4 ± 0.1 ^a^	1.95 ± 0.02 ^a^
	3	14.6 ± 0.3 ^a^	120.9 ± 8.2 ^a^	81.7 ± 2.4 ^a^	99.0 ± 0.1 ^b^	N.D.	4.05 ± 0.0 ^b^	3.61	4.4 ± 0.1 ^a^	1.74 ± 0.02 ^b^
	4	14.2 ± 0.9 ^a^	116.9 ± 2.1 ^a^	82.6 ± 0.6 ^a^	98.6 ± 0.0 ^c^	N.D.	4.95 ± 0.0 ^a^	3.59	4.1 ± 0.2 ^a^	1.66 ± 0.03 ^c^
UF-3K	1	14.1 ± 2.3 ^a^	107.9 ± 4.2 ^a^	80.9 ± 1.1 ^a^	99.6 ± 0.1 ^a^	N.D.	3.60 ± 0.0 ^b^	3.61	3.9 ± 0.1 ^a^	1.47 ± 0.02 ^d^
	2	14.2 ± 1.6 ^a^	101.0 ± 5.6 ^a^	78.0 ± 0.4 ^a^	98.5 ± 0.1 ^b^	N.D.	4.05 ± 0.0 ^a^	3.63	4.0 ± 0.1 ^a^	1.94 ± 0.00 ^a^
	3	14.8 ± 1.7 ^a^	109.8 ± 12 ^a^	73.9 ± 0.2 ^a^	98.7 ± 0.1 ^b^	N.D.	4.05 ± 0.0 ^a^	3.61	4.1 ± 0.1 ^a^	1.74 ± 0.02 ^b^
	4	14.1 ± 0.6 ^a^	97.2 ± 0.0 ^a^	75.1 ± 3.4 ^a^	97.8 ± 0.1 ^c^	N.D.	4.28 ± 0.2 ^a^	3.57	4.0 ± 0.2 ^a^	1.62 ± 0.04 ^c^

^1^ Not detected. ^a,b,c,d^ When the quality characteristics of rice wine with the same treatment have significant differences at *p*-value less than 0.05, homogeneous groups in each variable are identified by the same superscript letter according to the LSD test. APC: Aerobic plate count.
